# An e-Prehabilitation System of Care for Teenagers and Young Adults Diagnosed With Cancer: Protocol for a Qualitative Co-Design Study

**DOI:** 10.2196/10287

**Published:** 2018-09-12

**Authors:** Lisa McCann, Kathryn A McMillan, Christopher Hewitt

**Affiliations:** 1 Digital Health & Wellness Group Department of Computer and Information Sciences University of Strathclyde Glasgow United Kingdom; 2 Beatson West of Scotland Cancer Centre NHS Greater Glasgow & Clyde Glasgow United Kingdom

**Keywords:** digital health, human factors, co-design, prehabilitation, teenagers and young adults, cancer, mobile phone

## Abstract

**Background:**

A diagnosis of cancer in young adulthood can pose many different and unique challenges for individuals. The provision of adequate and appropriate information as well as care and support for teenagers and young adults at the time of diagnosis is central to their health care experience going forward. Moreover, appropriate and accessible information provision is critical to ensure that young individuals with cancer feel equipped and empowered to make decisions about, and be involved in, their treatment and recovery throughout their experience; this is a concept known as prehabilitation. As digital interventions and resources that support teenagers and young adults with cancer are an increasingly desirable part of health care provision, this study will focus on the development of an age- and population-appropriate electronic prehabilitation (e-Prehabilitation) system of care.

**Objective:**

We will conduct an exploratory, co-design research project that will inform the development of an e-Prehabilitation system of care to support teenagers and young adults diagnosed with cancer. A collaborative approach to data collection and prototype design will ensure that a patient-centered approach is embedded throughout.

**Methods:**

A qualitative, co-design study utilizing surveys, interviews, and focus group discussions is being conducted with teenagers and young adults, health care professionals, and technologists.

**Results:**

This research study is in progress; recruitment and data collection activities have commenced and findings are expected in early 2019.

**Conclusions:**

The findings of this study will have important implications for informing the future development and evaluation of an e-Prehabilitation system of care to support teenagers and young adults diagnosed with cancer.

**Registered Report Identifier:**

RR1-10.2196/10287

## Introduction

### Background

Although cancer is relatively rare in teenagers and young adults (TYA; individuals aged 15-24 years) [1], the incidence rates in the United Kingdom have increased by around one-fifth over the last decade [1]. In the United Kingdom, from 2009 to 2011, 2234 new cases of cancer were diagnosed among TYA. The diagnostic profiles of cancers in TYA differ from those in adults, with lymphomas being the most common group of cancers diagnosed in individuals aged 15-24 years [1]. Collectively, lymphomas, carcinomas, and germ cell tumors account for more than half of the total number of diagnoses of cancer in the 15-24-year-old population [1].

A diagnosis of cancer poses a range of physical, financial, and psychosocial challenges for young adults and their families [2-4], including disruption of education or career [5], family life [6], self-esteem or identity [7], peer and sexual relationships, and body image [8-10]. There is also a need to manage the side effects of treatment, both short and long term, and the possible impacts on future fertility [11-13]. These various challenges are often compounded by the developmental stages and changes that accompany young adulthood [14]. Thus, a diagnosis of cancer in young adulthood can pose many different and unique challenges for these individuals. As a consequence, there is a significant body of research that recognizes the importance of providing adequate and appropriate information as well as care and support to teenagers, young adults, and their families at the time of diagnosis [15-17]. Such information provision is critical in ensuring that young individuals feel equipped and empowered to make decisions about, and be involved in, their treatment and recovery, thereby enhancing a sense of mastery and control or self-efficacy [18].

There is an increasing interest in, and growing momentum regarding, the concept of “positive psychology” in the context of cancer [19]. Positive psychology includes building and strengthening the resilience of patients and their families following a diagnosis of cancer, adoption of coping strategies, and utilization of strengths based on assessments and interventions [20]. A key component of the positive psychology process is the proactive anticipation of challenges, which are likely to be encountered following a diagnosis of cancer and associated treatments to ensure that young individuals and their families can be equipped with information and effective coping strategies prior to the commencement of treatment, rather than during or after treatment, wherein a deficit or reactive model may have to be implemented [21].

The delivery of health-related interventions during the period between a patient’s diagnosis and treatment commencement is known as prehabilitation [22]. Typically delivered during the intervening period between a patient receiving a diagnosis and his or her treatment initiation, strategies tend to be implemented to maximize an individual’s fitness and adaptation, ultimately to have a positive impact on survival and associated patient-reported outcomes and coping [23-30]. The application of prehabilitation strategies is becoming more evident within health care, with a growing evidence base regarding their benefits for patients undergoing surgery [29,31], such as coronary artery bypass [32].

However, in the context of cancer care, prehabilitation is a relatively new and emerging concept. Against this backdrop, following a review of noncancer prehabilitation literature, cancer prehabilitation has recently been defined as follows:

...a process on the continuum of care that occurs between the time of cancer diagnosis and the beginning of acute treatment, includes physical and psychological assessments that establish a baseline functional level, identifies impairments, and provides targeted interventions that improve a patient’s health to reduce the incidence and the severity of current and future impairments, and provides targeted interventions that improve a patient’s health to reduce the incidence and the severity of current and future impairments [22].

In a review on cancer prehabilitation [22], it was concluded that opportunities exist for prehabilitation interventions to help support individuals with cancer. Such interventions may improve physical and psychological health outcomes, increase the number of potential treatment outcomes, and reduce direct and indirect health costs attributed to cancer. However, the paucity of prehabilitation research, developed interventions, and associated packages of care within this context indicates that there is an actual need to identify the best interventions for various groups of individuals affected by cancer [22]. Developing tailored prehabilitation interventions and associated packages of care for particular patient groups is critical to ensure that they are responsive to and meet the particular needs of these patient groups. Ultimately, doing so may help increase the rates of compliance with treatments, consequently having a positive impact on treatment survival outcomes [22]. Despite the potential benefits of an established prehabilitation program, there is a notable paucity of information that focuses on TYA with cancer within this context.

New interventions should be designed and developed in collaboration with the target population to help ensure their success. Evidence-based co-design in which an individual’s experience is explored and collated to help develop service improvements [33] is one recognized way of ensuring a person-centered approach for care. In addition, young individuals are now digital natives [34]; thus, digital resources to support TYA with cancer are increasingly desirable. In a recent service evaluation survey on TYA with cancer at a clinical site in one area of the United Kingdom, TYA were responsive to the potential provision and development of digital resources that will support them during their cancer experiences [34]. In another service development initiative in the United Kingdom, a digital pathway, which is known as the Integrated Assessment Mapping (IAM) portal, was recently developed by the University Hospitals Bristol National Health Service (NHS) Foundation Trust and the TYA South West Cancer service. The project aimed to provide emotional and clinical support to TYA cancer patients using a holistic, age-appropriate digital platform [35]. Via a cocreation approach, 3 interconnected services were developed following engagement sessions with the TYAs. The 3 services included a TYA website, the IAM website and mobile app, and the SWIMMS patient database. Collectively, these 3 services provide support to patients based on their self-identified needs; additionally, the clinical care team and service providers are able to better identify the support needs of TYA with cancer based on the information provided by the patients themselves [35]. The IAM project highlights that it is possible to co-design, develop, and integrate an eHealth platform to provide support to TYA diagnosed with cancer.

Thus, collaborative engagement; prioritization of TYA’s experiences and needs; and the development of a suitable, effective, and appropriate digital health solution are the central tenants of this study. As the current evidence base for prehabilitation care is limited, particularly for TYA with cancer, this is the right time to develop an evidence-based and experientially informed system of care for this patient population.

### Study Objectives

This exploratory research project will inform the development of an electronic prehabilitation (e-Prehabilitation) system of care to support TYA diagnosed with cancer. To achieve this, there are four overarching research objectives: (1) understand the needs of TYA with cancer at the time of diagnosis; (2) understand the potential role of eHealth solutions to assist in the prehabilitation care offered to TYA with cancer by health care professionals (HCPs); (3) identify appropriate technologies and technological platforms to support the delivery of an e-Prehabilitation system of care, and (4) generate the content of a prototype e-Prehabilitation system of care for use by HCPs and TYA diagnosed with cancer.

## Methods

### Study Design

This study draws on two main conceptual frameworks to ensure that the intervention is developed appropriately. First, the study draws on the Medical Research Council (MRC) Framework for the development, evaluation, and implementation of complex interventions to improve health [36]. Drawing on Stage I of the MRC Framework, activities in this study will focus on engaging with TYA and professionals working in health care or technologists working with digital health solutions and innovations in industry or the NHS and developing the theory to inform the development of the intervention. Theoretically, this study will draw on the Behavior Change Wheel as this provides a framework for understanding behavior as an enabler for behavior change interventions [37].

### Ethics Approval

The ethical aspects of this study were approved by the Yorkshire and the Humber–Bradford Leeds research ethics committee, and it was endorsed by the lead author’s University ethics committee (Research Ethics Committee Reference:17/YH/0352). The study has also received Research & Development approval from the relevant NHS Board in the United Kingdom.

### Eligibility Criteria

The eligibility criteria for participation in the study are provided in Table 1. Although teenage and young adulthood is defined as the period between 15-24 years of age, 16-26 years is the age range for referrals for TYA to access services at the partnering clinical site. TYA and HCPs who fit the inclusion criteria and provide informed consent will be eligible to participate in the study.

**Table 1:** Eligibility criteria of the study participants.

Participant group	Inclusion criteria	Exclusion criteria
Teenagers and young adults (TYA)	Young individuals aged 16-26 years TYA who were diagnosed with cancer up to 3 years, but no less than 4 weeks prior to participation in the study. TYA may be undergoing acute anticancer treatments and therapies and maintenance treatments, or may be considered to have completed all treatments during the study period and up to 3 years postdiagnosis Receiving or received services from the National Health Service in Scotland or partner cancer principal treatment center Able to participate in data collection activities Able to provide informed consent Able to communicate sufficiently well in English	Teenagers younger than 16 years Young adults older than 26 years TYA without a cancer diagnosis TYA newly diagnosed with cancer (within the last 4 weeks) TYA who received a diagnosis of cancer more than 3 years ago Unable to provide informed consent Unable to communicate sufficiently well in English
Health care professionals	Members of the TYA cancer team or multidisciplinary team involved in the provision of care or services to TYA with cancer Have experience of working with TYA who have or have had a diagnosis of cancer Able to provide informed consent Able to communicate sufficiently well in English	Unable to provide informed consent Unable to communicate sufficiently well in English
Technologists or digital health professionals	Professionals with experience of working within the digital health space within the National Health Service or industry or academia Able to provide informed consent Able to communicate sufficiently well in English	Unable to provide informed consent Unable to communicate sufficiently well in English

### Recruitment

#### Teenagers and Young Adults

##### Direct Recruitment

**Figure 1:**
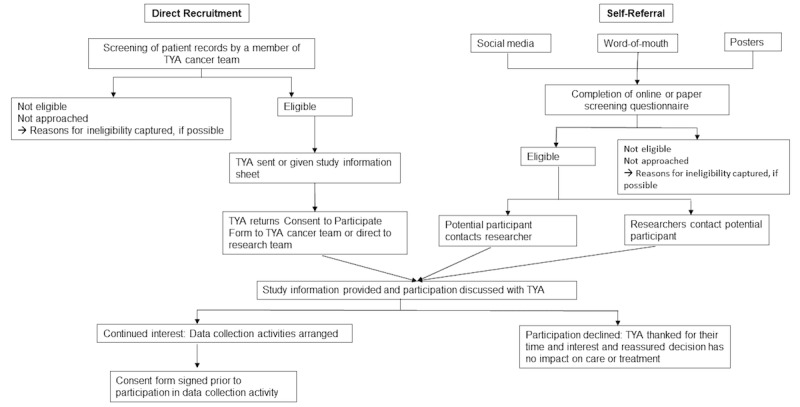
Flowchart of the recruitment. TYA: teenagers and young adults.

Patient databases will be screened for TYA who meet our inclusion criteria by members of the TYA cancer team at the recruiting site (Figure 1). A staff member will first approach potential participants in person or via email to introduce the possibility of participating in the study and to provide the study information material. Potential participants will be asked to complete or provide permission for the proxy completion of a consent-to-be-contacted form (Figure 1) to indicate their agreement for passing on their contact details to the research team. Only then will the research team contact TYA to further discuss participation in the study. If possible, we will capture top-level details of ineligibility for invitations to participate from the databases in partnership with the TYA cancer team.

##### Self-Referral

We will adopt a variety of self-referral strategies to provide TYA with opportunities to participate in the study. We will circulate study invitations in the form of recruitment advertisements or posters or postcards and display these at the recruiting principal treatment center and various other environment-appropriate locations throughout Scotland. Social media will be utilized to further increase the opportunities to recruit TYA; where appropriate, we will also post digital versions of the recruitment advertisements. Study-specific Twitter, Facebook, and email accounts have been created. We will also circulate information to other relevant support organizations working directly with TYA with cancer. If necessary, paid advertisements on social media and in the local press will be used to aid recruitment.

Potential TYA participants will be directed to a web-based screening questionnaire to establish their eligibility for study participation. The web-based screening questionnaire will also gather information regarding eligibility criteria that were not met. Thus, the research team can track reasons for non-participation at the screening stage.

#### Health Care Professionals

Researchers will directly invite HCPs such as consultant oncologists, psychologists, allied health professionals, social workers, specialist nurses, ward nurses, and youth support professionals to participate in the study. This will help ensure appropriate multidisciplinary participation in the study.

#### Technologists or Digital Health Professionals

Key individuals with a responsibility for digital health within the NHS, industry, and academia will be directly approached by members of the research team to participate in the study.

### Participant Information and Informed Consent

TYA and professionals will receive information about the study before consenting to participate in any data collection activities (Figure 1). TYA participants will receive a written information sheet in age-appropriate language and a link to a video and audio information sheet created specifically for this project. The video clip will be made available via the project website. Professionals will receive a written participant information sheet distributed via email or hard copies. Potential participants (TYA and professionals) will have at least 24 hours to familiarize themselves with the study information and to decide whether or not they wish to participate. TYA and professionals will have the opportunity to ask questions prior to signing the consent form or recording verbal consent. In instances where it is not possible to obtain a written consent from the participants (eg, when conducting telephone interviews), verbal consent will be obtained instead at the start of the audio recording.

### Incentives

The indirect benefits of taking part in this study for TYA and professionals are sharing experiences and contributing to the development of the design of a new digital health intervention. TYA will receive a study-specific certificate of participation from the research team, which may benefit their portfolio for further education and/or job applications. TYA will be reimbursed for their travel expenses for attending any data collection activities away from their home, and refreshments will be provided during any group data collection activities.

### Data Collection

**Figure 2:**
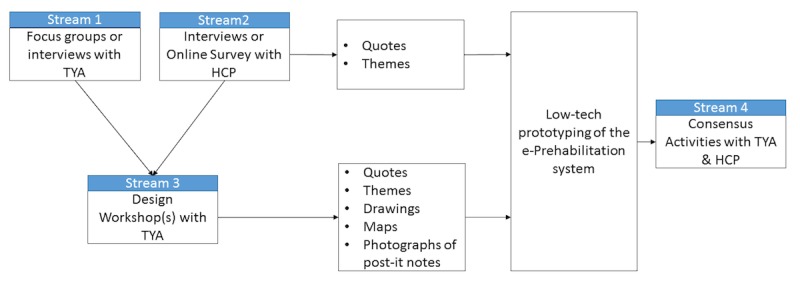
Flowchart of the different stages of data collection activities and outputs. TYA: teenagers and young adults; HCP: health care professionals.

Throughout the data collection activities, we will utilize TYA’s position of being an expert on the general knowledge of experiences of young TYA at the time of cancer diagnosis, based on their own experiences. By doing so, we will empower TYA to participate as expert representatives of others as well. There are 4 distinct streams of data collection in this study, as outlined in Figure 2.

#### Stream 1: Focus Group Discussions or Individual Interviews With Teenagers and Young Adults

We will conduct focus group discussions or individual interviews with TYA with a history of cancer diagnosis to develop an understanding of the issues they faced after the diagnosis. The focus group discussions will be conducted if at least three participants are available for discussion. If we are able to conduct focus group discussions, we aim to recruit up to 20 TYA who meet the inclusion criteria to participate in the first stage of data collection. We will conduct up to four separate focus group discussions with 3-7 participants each. However, if engagement levels for focus group discussions are low and we are unable to meet the minimum number of required participants (n=3) to conduct the discussions, we will conduct a series of 1-1 interviews instead of or alongside focus group discussions for the first stage of data collection. In this case, we will recruit up to 10 TYA who meet the inclusion criteria. Individual interviews will last up to 60 minutes and focus on the topics outlined above.

#### Stream 2: Interviews or Web-Based Survey with Health Care and Digital Health Professionals

Stream 2 will run concurrently with stream 1. In stream 2, the professionals will be invited to participate in one-off individual interviews to explore their preferences for the content and delivery of the e-Prehabilitation system of care, including the technology platform to be used and their requirements for the prehabilitation care resources and materials that should be included in the intervention. Interviews will last approximately 30 minutes and will be conducted face-to-face or via telephone based on participants’ preference and audio recorded. To enhance opportunities for participation, we will also provide the professionals with the opportunity to complete a short web-based survey. The link to the web-based survey will be distributed via email.

#### Stream 3: Design Workshops

Drawing on data gathered in streams 1 and 2, the research team will explore with TYA the technology platform to be used as well as the type, nature, and focus of the content of the material contained in the system and their requirements for this system. Design workshops will emphasize the need for technology that is not just designed for illness and medical activities. This requirement has been expressed by teenagers with a chronic medical diagnosis previously [38]. Participatory design activities for participants during stream 3 are shown in Table 2.

#### Stream 4: Consensus Activities

The data generated from streams 1-3, in combination with the findings from the literature, will be considered collectively to develop a low-fidelity prototype. In stream 4, we will endeavor to seek feedback from participants on this low-fidelity prototype. To do this, we will provide physical (as in face-to-face group or individual discussions) and/or electronic (as in a web-based discussion forum or survey) environments for participants (TYA and professionals) to access and comment on the low-fidelity prototype. If the interest from participants warrants a further face-to-face meeting, a session will be arranged to present the prototype to TYA and professionals together. However, if we find it difficult to bring people together physically, we will distribute an electronic version of the prototype (eg, via PDF files of wireframes of the suggested content) via email or secure transfer and ask for comments and feedback. Participation in stream 4 will be optional for both TYA and professional participants.

**Table 2:** Participatory design activities for stream 3.

Topics	Purpose	Tool kit of the methods (will be selected or adapted as appropriate)	Data output
Content—Nature and focus	To gather some ideas about the type of information displayed and collected	Researchers present a “Tree of ideas” made up of tag-clouds of themes generated from the focus group discussions or interview responses from streams 1 and 2. Participants rate ideas as “keep,” “lose,” or “change” using color-coded post-it notes. Verbal discussions and/or blank paper sheet for items labeled as “change,” participants suggest how the item should be changed for being rated as “keep.” Participants rank ideas labeled as “keep” by priority using sequential numbers.	Voice recording, text (annotations), photographs of post-it notes
Content—Visual designs	To capture some ideas about how the information might look	Researchers provide A4 paper sheets with blank smartphone mock-ups. Warm-up activity on paper prototyping. Researchers provide examples of existing electronic cancer information tools via iPad (eg, Integrated Assessment Mapping website, websites and materials from cancer charities, video game: Re-Mission). Participants formulate ideas or write descriptions of how the information could be visually presented on a smartphone app or website.	Drawings, text (annotations), voice recordings
Functions and features	To get a view of what the technology looks like and some of its properties	Researchers provide A4 paper sheets with blank smartphone mock-ups. Participants formulate functions and features or write descriptions (what functions or features and how they work).	Drawings, text (annotations), voice recording
Contextual enquiry of technology use	To capture ideas and preferences on the type of digital health technology and how and when it would be used	Research team provides 3 printed maps of a fictional town with images of typical locations and buildings where teenagers and young adults might find themselves. Each map will represent a point in time of the cancer journey (Diagnosis, Treatment, and Survivorship). Color-coded stickers, pens, and post-it notes will also be provided. Participants will indicate on the map which type of information they would access or seek and where they would use a digital health intervention to do so.	Maps, text (annotations), photographs of post-it notes, voice recordings

### Demographic and Clinical Questionnaire

We will ask participants to complete a questionnaire to obtain the demographic and clinical (TYA only) characteristics of the participants on the day of the focus group discussions or interviews or web-based survey prior to participating in data collection activities. All data entries will remain anonymous.

### Data Analysis

#### Focus Group Discussions, Interviews, and Web-Based Survey

Focus group discussions or interviews with TYA with cancer and professionals will be audio recorded and transcribed verbatim. Transcripts will be merged with field notes and outputs of brainstorming activities. During the analysis, two researchers will draw upon the research objectives and identify and develop themed categories to guide the data analysis. We will use NVivo (version 12; QSR International; Australia), a qualitative data analysis software package, to support these activities.

Thematic analysis is a useful approach for answering questions about the salient issues for a particular group of respondents or for identifying typical responses [39]. For reliability and validity purposes, two researchers will code a subsample of transcripts and field notes separately and then cross-check them together.

#### Design Workshops

Design activities will be audio recorded to capture the discussions and reflections about design processes and products. It is less likely that audio recordings from design workshops will be transcribed due to the expected levels of background noise. However, the research team will listen to them as an aide-memoire. We will take photographs of design sheets and maps that include post-it notes and stickers to avoid losing the sticky pads when being transported for analysis. With participants’ permission, we will take photographs of group work interactions during streams 3 and 4.

As summarized in Table 2, the data output of the design workshop will comprise a number of different data types (texts, maps, and drawings). Two researchers will independently code the design ideas based on a predefined and piloted coding template using all available data sources. The coding of design ideas will be cross-checked between the researchers, and disagreement will be resolved by a third researcher. Independently, the two researchers will select the “single best” design idea from each group or individual work that will be considered for prototyping the e-Prehabilitation system.

### Data Management

#### Participant Confidentiality

Personal data recorded on all documents will be considered as confidential, and participants will be allocated a unique study number by the research team for reporting purposes. Participants’ personal details will not be recorded on any interview transcripts or surveys; only their designated unique study number will be included in these documents. Any identifiable information captured during the interviews will be anonymized during the transcription process. The participant identification key, which links the unique study number with the participants’ name, will be stored in a separate location to participants’ personal data.

#### Data Storage and Disposal

##### During the Study

All data will be stored in locked filing cabinets at the lead author’s institution. Personal data will be stored in a separate filing cabinet from anonymized hard copies of the data. Focus group discussions or interviews will be digitally audio recorded on a password-protected recording device. All transcripts will be anonymized and stored in the secure, shared network of the lead author’s institution on password-protected computers. Only authorized members of the research team will have access to the network drive and locked filing cabinets.

##### After the End of the Study

Personal data will be stored for 6-12 months after the study has ended to allow the dissemination of research findings to study participants. Anonymous research data will be stored for 10 years after this study has ended. After 10 years, the data will undergo a review process conducted by the University’s Research Data Management and Sharing Team that will decide whether the data will remain in long-term storage or deleted.

## Results

The recruitment and data collection for this study commenced in February 2018, and results will be submitted for peer review upon completion of data collection and analysis. The project is expected to end in early 2019.

## Discussion

The current protocol describes a collaborative co-design study designed to focus on the development of an e-Prehabilitation system of care for TYA with cancer as well as its future use within the current service delivery models. The study design is appropriate for the development of an intervention, utilizing multiple perspectives and data collection methods. The findings from this study will have important implications for informing the future development of an e-Prehabilitation system of care to support TYA diagnosed with cancer.

## Abbreviations

e-Prehabilitation: electronic prehabilitation
HCP: health care professional
IAM: Integrated Assessment Mapping
NHS: National Health Service
MRC: Medical Research Council
TYA: teenagers and young adults
